# Plasma Autoantibodies Against Neurodegeneration-Related Antigens in Dementia and Elevated Chi3Li Autoantibodies in Mild Cognitive Impairment

**DOI:** 10.3390/biom16040518

**Published:** 2026-03-31

**Authors:** Gabriela Kocurova, Zuzana Svabenska, Jan Klaschka, Ales Bartos, Jan Ricny

**Affiliations:** 1 Experimental Neurobiology Program, National Institute of Mental Health, 250 67 Klecany, Czech Republic; gabriela.kocurova@nudz.cz; 2 Institute of Molecular Genetics of the Czech Academy of Sciences, 142 20 Prague, Czech Republic; zuzana.svabenska@img.cas.cz; 3 Institute of Computer Science of the Czech Academy of Sciences, 182 00 Prague, Czech Republic; klaschka@cs.cas.cz; 4 Department of Neurology, Third Faculty of Medicine, Charles University, 100 00 Prague, Czech Republic; 5 Department of Neurology, University Hospital Kralovske Vinohrady, 100 00 Prague, Czech Republic

**Keywords:** autoantibodies, neurodegenerative diseases, dementia, fluid biomarkers, neuroinflammation, astroglial activation

## Abstract

Systemic autoimmunity plays an important role in pathogenesis of neurodegenerative diseases. The objective of our study was to explore the seroprevalence of naturally occurring autoantibodies (Aabs) targeting a panel of 14 antigens broadly involved in neurodegenerative diseases such as Alzheimer’s Disease, Parkinson’s Disease, frontotemporal dementia, and vascular dementia. Commonly associated proteins with underlying neuronal pathology of the brain include amyloid-beta (Aβ), tau, alpha-synuclein (α-syn), TDP-43, and FUS. Proteins associated with glial and astrocytic involvement—TREM2 and Chi3Li; proteins related to myelin damage and axonal degeneration—light neurofilaments (NFL), myelin basic protein (MBP); synaptic loss reflected by neurogranin (NRGN), a marker of neuronal injury—neuron specific enolase (NSE); and markers of disturbed calcium homeostasis—VSNL1 and neuroinflammation—MCP-1. Presence and levels of plasma IgG against these antigens were examined using enzyme-linked immunosorbent assay (ELISA) method in patients with dementia, patients with mild cognitive impairment (MCI), and healthy age-matched controls. Aabs against all selected antigens were detected across all groups, including healthy control, with varied seroprevalence levels. For the first time, we report the presence of anti-FUS, anti-TREM2, anti-NRGN, anti-VSNL1, anti-NSE, and anti-MCP1 Aabs. Elevated anti-Chi3Li Aabs in individuals with MCI indicate a disease-associated immune signature linked to early neurodegenerative processes. Overall, these results provide evidence of systemic immune activation accompanying neurodegeneration, underscore the complexity of immune involvement, and highlight the importance of targeting multiple pathological pathways in future immunomodulatory strategies.

## 1. Introduction

Incidence of neurodegenerative diseases (NDDs) is rising at an unprecedented rate. NDDs such as Alzheimer’s disease (AD), Parkinson’s disease (PD), dementia with Lewy bodies (DLB), frontotemporal lobar neurodegeneration (FTD) or amyotrophic lateral sclerosis (ALS) increase with the global population aging. AD is the most common form of dementia, affecting more than 50 million people worldwide accounting for 60–80% of all dementia cases [1]. PD, the second most prevalent neurodegenerative disorder, shows a sharp increase in incidence after age 50, with an estimated global prevalence of 21.7 to 30.1 million people [2]. Other neurodegenerative conditions including ALS, Huntington’s disease (HD) and FTD are also becoming more widespread. This growing burden places immense pressure on healthcare systems and society. These diseases share a common pathological pathway which leads to neurodegeneration, loss of neurons in particular parts of the brain and cognitive and behavioral dysfunction accompanied with protein misfolding and aggregation [3,4]. Until recently, treatment was only symptomatic; nevertheless, with recent advancement in the field, disease-modifying therapy has proven efficient [5]. Future progress may lead to targeted or multi-targeted therapies capable not only of slowing but potentially halting, reversing, or preventing disease progression [6,7]. Many NDDs have a long prodromal phase with no, or very subtle, symptoms which are often undetectable. Large variability between molecular pathology and clinical phenotype due to multiple impaired pathways together with frequent occurrence of co-pathologies makes the diagnostic process particularly challenging. Ultimately, this heterogeneity affects patient stratification in clinical trials and outcomes, and complicates investigations into effective implementation of disease-modifying therapies. [8,9]. Therefore, strategies that enable early detection of a neurodegenerative process prior to the onset of symptoms, and approaches that address both disease-specific and non-disease-related pathologies, will be critical to curbing the projected rise in dementia cases [10]. Perhaps subtyping the NDDs according to their biological and biochemical characteristics rather than clinical definitions will ease the definition of disease phenotypes. Currently, cerebrospinal fluid (CSF) analysis and neuroimaging represent the primary diagnostic tools. However, there is a need for a low-cost diagnostic with high accuracy for screening large populations during the preclinical stages of disease. Biomarkers circulating in blood have the advantage of being cost- and time-effective as blood collection and analysis cost less than 10% of CSF-based or neuroimaging-based diagnostics. An ideal biomarker should identify diagnostically or prognostically homogenous patient groups, predict disease progression, support patient recruitment for clinical trials and enable treatment response monitoring [11,12]. Substantial progress in fluid biomarker development has been achieved in AD, where validated measures of amyloid-beta (Aβ) and tau are now widely used in both research and clinical practice. In contrast, reliable biomarkers for other NDDs, such as those involving alpha-synuclein (α-syn), TAR DNA-Binding Protein 43 (TDP-43), or non-AD tauopathies, remain limited. There is also a growing need for biomarkers that reflect broader biological processes relevant to disease, including neuroinflammation and vascular dysfunction. Together, these markers should provide a more complete picture of the molecular and pathophysiological changes that drive neurodegeneration.

Naturally occurring autoantibodies (Aabs) are a subset of antibodies which are directed to self and altered self components. They are present in newborns in the absence of external antigenic stimulation [13] reflecting early exposure to the endogenous self structures [14]. Aabs are increasingly recognized as potential biomarkers for NDDs and are under investigation for their diagnostic, prognostic, and therapeutic potential [15,16]. They contribute to immune surveillance and clearance mechanisms by targeting neoepitopes and aggregated or misfolded proteins [17]. Although Aabs are unlikely to enter intracellular compartments, they may facilitate the phagocytosis of dying cells and contribute to their removal, while also monitoring the extracellular space to inhibit the intercellular propagation of pathological protein species. While Aabs are typically present in healthy individuals, altered concentrations have been reported in patients with NDDs such as AD, DLB, FTD and vascular dementia (VD) as well as other neurological disorders [11,18,19,20]. Protein aggregation is a common pathological feature of many NDDs and plays a key role in their diagnosis and classification [4]. Changes in both the concentration and binding affinity of Aabs targeting α-Syn, Aβ and tau have been documented across these disorders, suggesting that impaired immune clearance of pathological proteins may contribute to neurodegenerative processes [21,22]. The exact contribution of Aabs to NDDs mechanisms is not fully understood. While some studies suggest that Aabs may exert protective effects, others indicate potential pathogenic roles depending on their specificity, concentration, and affinity [23,24,25]. Supporting the therapeutic potential of antibody-based strategies, preclinical studies have demonstrated beneficial effects of passive immunization in animal models [26]. More recently, monoclonal antibodies targeting Aβ, such as lecanemab and donanemab, have shown promising results in clinical trials and are currently approved for use in patients with mild cognitive impairment (MCI) [27,28,29,30].

To investigate immune activation associated with neurodegeneration, we analyzed plasma samples from patients with various NDDs. Our aim was to characterize humoral immune response to antigenic moieties released from affected brain regions and to determine whether specific Aab signatures could differentiate patients from healthy controls. Using enzyme-linked immunosorbent assays (ELISAs), we performed discovery analysis to identify novel Aabs associated with neurodegenerative pathology. Several in-house ELISA assays were developed and optimized to detect circulating Aabs. Plasma Aabs positivity against 14 target antigens was quantified across patients with AD, FTD, PD, VD, MCI, and healthy controls.

## 2. Materials and Methods

### 2.1. Human Participants, Patients Consent, Sample Collection and Study Design

The research was approved by the Ethics Committee of National Institute of Mental Health, Klecany, Czech Republic, and it was conducted according to the Declaration of Helsinki and the Act no. 129/2003 and 130/2003 of the Czech Republic.

Samples from patients and healthy controls were obtained from a biobank created at Faculty Hospital Královské Vinohrady. All participants and witnesses signed an informed consent.

Plasma samples were collected from a total of 159 patients at the Department of Neurology or the Memory Clinic of Charles University. The cohort included 15 patients with VD, 10 with PD, 36 with FTD (including 2 bvFTD), 28 with MCI due to AD, 58 with AD, and 12 healthy individuals. The healthy control group consisted of age-matched participants with no prior history of NDDs, memory impairment, or autoimmune disease. As naturally occurring Aabs are commonly present in healthy individuals as part of physiological immune homeostasis, their detection in control samples was not considered indicative of pathological autoimmunity, but to establish baseline seroprevalence and enable comparison with patient groups. Collection was scheduled for 7–10 a.m., blood was drawn in a lithium-heparin tube from the antecubital vein. After a maximum time of 30 min, whole blood was centrifuged at 1000× *g* for 15 min at 4 °C to separate plasma. Plasma was then pipetted in small Eppendorf tubes. They were centrifuged at 10,000× *g* for another 15 min at 4 °C. Plasma was kept at −80 °C before assaying.

The cognitive functions of the patients were assessed using the Mini-Mental State Examination (MMSE, 0–30) [31,32]. Patients with mild cognitive impairment due to AD (MCI) were diagnosed according to the National Institute on Aging–Alzheimer’s Association (NIA-AA) criteria [33] and cerebrospinal fluid (CSF) biomarkers associated with AD pathology such as Aβ, total tau (t-tau), and phosphorylated tau (p-tau), were measured. Biomarker distributions in the MCI cohort were summarized using median and 25th–75th percentile. Patients with dementia due to AD (AD) were diagnosed based on the NIA-AA criteria [34].

### 2.2. Baseline Characteristics of the Cohort

Demographic characteristics of the study cohort are summarized in Table 1.

### 2.3. In-House ELISA Assays Development, Optimization and Testing

We developed a set of in-house ELISA assays to compare the levels of naturally occurring Aabs against neuronal, glial, and astrocytic markers. Recombinant human antigens were obtained from commercial suppliers as listed in Table 2. Manufacturer-reported purity was ≥95%, assessed by SDS-PAGE and/or HPLC. Proteins were supplied either lyophilized or frozen and were reconstituted according to the manufacturer’s instructions. Aliquots were prepared and stored at −80 °C to avoid repeated freeze–thaw cycles.

Complete list of antigens used is listed below (Table 2).

During assay development, representative cohort samples were tested at serial dilutions (1:50, 1:150, and 1:450) to verify dilution-dependent signal behavior and identify a working dilution within the assay’s dynamic range. A dilution of 1:100 was selected for final analyses as it provided optimal distinction between reactive and non-reactive samples while avoiding signal saturation observed at lower dilutions. Because no commercial antibody standard is available for most naturally occurring Aabs tested, the assay was designed as semi-quantitative. Binding solution (50 mM bicarbonate buffer pH 9.4/deionized water) was used for antigen coating. After a series of optimizations, the final antigen concentration of 0.5 µg/mL was determined. Antigens were diluted in the binding solution and plates (Corning^®^ 96 Well EIA/RIA Assay Microplate high binding surface, 3361, Corning Incorporated—Life Sciences, Kennebunk, ME, USA) were coated with 50 µL of the coating solution per well. Plates were left an hour on bench at room temperature and then incubated for 20–24 h overnight. Subsequently, plates were blocked with 1% BSA in PBST (10 mM Na-phosphate pH 7.4/100 mM NaCl/0.05% Tween 20), 200 µL per well, and incubated overnight at 4 °C. After blocking, wells were washed 3 times with PBST and dried in an incubator for 3 h at 37 °C. Plates were used for further analysis or vacuum-packed and stored in a freezer at −80 °C. Plasma was 100× diluted in the dilution solution (SeramunStab^®^STB, Heidesee, Germany). A total of 100 µL of the diluted sample was pipetted into the wells, incubated for 60 min at room temperature, and washed 4 times with 200 µL PBST solution. Dilution solution was used as a blank sample; uncoated control wells were tested for each sample to determine unspecific binding of the particular plasma. As a secondary antibody, a conjugate of Goat Anti Human IgG Fc Fragment specific (Jackson Immuno Research, cat.no.: 109-035-008, Jackson Immuno Research Laboratories, Inc., West Grove, PA, USA) with horseradish peroxidase diluted 1:15,000 in the dilution solution was used. A total of 100 µL of the diluted conjugate was pipetted into each well and incubated for 1 h at room temperature, then washed 4 times with 200 µL PBST. Then, 100 µL of TMB chromogenic substrate (SeramunBlau^®^ fast, Seramun Diagnostica GmbH, Heidesee, Germany) was added to each well. The plate was covered to be protected from light and incubated for 15 min at room temperature on a shaker. After the incubation, reaction was stopped by adding 100 µL 0.4 M H_2_SO_4_ to each well. Plates were placed in a spectrophotometer Elisa Reader Multiskan EX (Thermo Scientific, Waltman, MA, USA) and the absorbance measurements were taken at 450 nm; 650 nm wavelength was used as a reference. Each plate included blank wells (buffer only), antigen-free wells for background determination, and the pooled reference plasma standard. All samples and controls were measured in duplicate.

### 2.4. ELISA Assays Quality Control and Data Categorization

For each plate and antigen, a background signal was determined using antigen-free control wells (ODc). Acquired optical density values were background-corrected to account for inter-plate variability, a pooled standard plasma sample (prepared from >50 cognitively unimpaired donors) was included in duplicate on every plate. Signal-based assay sensitivity thresholds were defined at the reporting dilution. The limit of detection (LOD) was defined as the mean signal of negative control wells plus 3 standard deviations, and the limit of quantification (LOQ) was defined as the mean negative signal plus 10 standard deviations. ODc values of uncoated control wells were subtracted from sample OD values and results were normalized to a standard plasma sample.

Intra-assay variability was assessed using replicate wells of patient samples within plates and was determined at 5.36–9.30% range. The inter-assay variation between plates as an indicator of the reproducibility was calculated from 4 measurements for each antigen and was under 20% each time.

Assay specificity was evaluated during assay development using antigen-free wells to confirm antigen-dependent signal detection, and background signals from uncoated wells remained consistently low across plates, confirming that measured signals primarily reflected antigen-specific antibody binding.

As maximal OD responses differed between antigens (approximately 1.0–2.5), normalized background-corrected signals (OD) were categorized into predefined semi-quantitative classes (0, 0+, +, ++, +++) to facilitate clearer graphical representation and comparison across antigens (Table 3). The scoring system was selected based on the assay sensitivity thresholds, where the 0+ category corresponds to signals above the LOD and the + category corresponds to signals above the LOQ.

### 2.5. Statistical Analyses

Statistical analyses data were performed using R statistical software version 4.4.3 and graphs were created in GraphPad version 10.6.1 [35]. Descriptive statistics like means, standard deviations, medians, quartiles, and percentages were used to summarize the data. The Pearson chi-square (χ^2^) test was used for comparison of sex distributions in different groups. Differences in semiquantitative or quantitative parameters between multiple groups were tested by non-parametric Kruskall–Wallis test, followed by post hoc Mann–Whitney tests of pairwise differences. Moreover, effect sizes for group pairs [36] were calculated using R package rstatix [37]. To account for multiple comparisons, *p*-values were adjusted using the Benjamini–Hochberg false discovery rate (FDR) procedure. This method controls the expected proportion of false discoveries among significant results when multiple hypotheses are tested simultaneously. After correction, *p*-values less than 0.05 were considered statistically significant. A small subset of participants had missing demographic data due to incomplete clinical records. All analyses were conducted using available data.

## 3. Results

### 3.1. Clinical Characteristics

Significant differences between diagnostic groups were observed for both age (Kruskal–Wallis test, *p* = 0.025) and sex distribution (χ^2^ test, *p* = 0.037).

Investigation of the χ^2^ components indicated that this difference in sex distribution was primarily driven by a higher proportion of male participants in the FTD group. Age differed significantly across diagnostic groups (Kruskal–Wallis test, *p* = 0.025). Pairwise comparisons indicated that the AD group was significantly older than the FTD, VD, and MCI groups. No significant age differences were observed among the remaining diagnostic groups.

CSF biomarker characterization of the MCI cohort showed median and 25th–75th percentile (Q_25_–Q_75_) levels of Aβ 568 (427–723) pg/mL, t-tau 514 (232–670) pg/mL, and p-tau 59 (33.5–77.8) pg/mL.

### 3.2. Overview of Autoantibody Profiles Across Neurodegenerative Diseases

**Figure 1:**
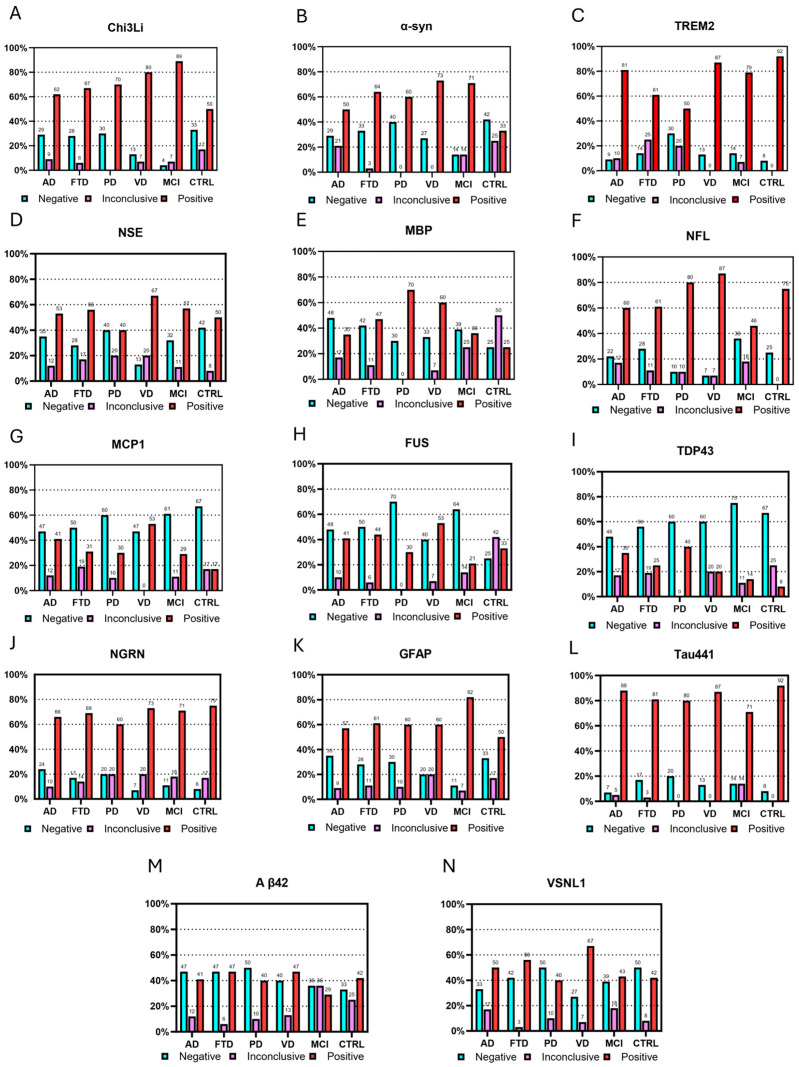
Seroprevalence of autoantibodies against neurodegeneration-related antigens across diagnostic groups, illustrating differences in antibody detection frequencies between patients and healthy controls. Chi3Li (**A**), anti-α-syn (**B**), anti-TREM2 (**C**), anti-NSE (**D**), anti-MBP (**E**), anti-NFL (**F**), anti-MCP-1 (**G**), anti-FUS (**H**), anti-TDP-43 (**I**), anti-NGRN (**J**), anti-GFAP (**K**), anti-Tau441 (**L**), anti-Aβ42 (**M**), and anti-VSNL1 (**N**) autoantibodies and the percentage of positive patients within the diagnostic group are shown; inconclusive results represent all the samples with optical density indistinguishable from 0 (0+). AD, Alzheimer’s disease; FTD, frontotemporal lobar neurodegeneration; PD, Parkinson’s disease; VD, vascular dementia; MCI, mild cognitive impairment; CTRL, control group. Chi3L1, chitinase-3-like protein 1; α-syn, alpha-synuclein; TREM2, triggering receptor expressed on myeloid cells 2; NSE, neuron-specific enolase; MBP, myelin basic protein; NFL, neurofilament light chain; MCP-1, monocyte chemoattractant protein-1; FUS, fused in sarcoma; TDP-43, TAR DNA-binding protein 43; NRGN, neurogranin; GFAP, glial fibrillary acidic protein; Tau441, full-length tau protein; Aβ42, amyloid-beta 1–42 peptide; VSNL1, visinin-like protein 1.

Aabs against the 14 selected antigens were detected across all diagnostic groups, including healthy controls, with variable prevalence (Figure 1). Several Aabs, including those targeting NSE, NRGN, GFAP, and Tau441, were commonly observed across multiple groups, indicating widespread baseline autoreactivity. In contrast, distinct patterns were observed for selected antigens: Chi3L1 and α-syn Aabs showed higher prevalence in patients with MCI and several dementia groups, whereas TREM2 Aabs were more frequently detected in healthy controls and were reduced in most patient groups. Other antibodies, including those against MCP-1, FUS, and TDP-43, were less prevalent overall.

Clinical data of patients with dementia, patients with MCI, and cognitively healthy control individuals are shown in Table 2. Frequencies of immunoglobulin G (IgG) Aabs are depicted on the Figure 1.

### 3.3. Levels of Circulating Autoantibodies Across Neurodegenerative Diseases

**Figure 2:**
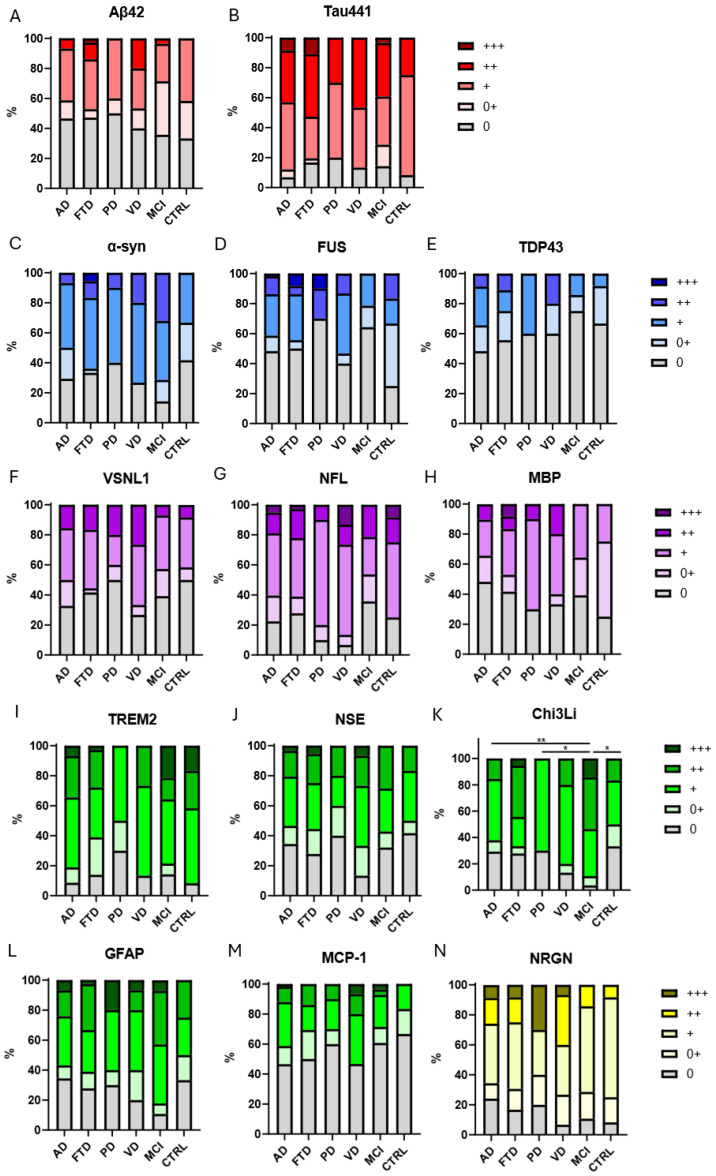
Relative levels of IgG autoantibodies against Aβ42 (**A**), Tau441 (**B**), α-syn (**C**), FUS (**D**), TDP-43 (**E**), VSNL1 (**F**), NFL (**G**), MBP (**H**), TREM2 (**I**), NSE (**J**), Chi3Li (**K**), GFAP (**L**), MCP-1 (**M**), and NGRN (**N**). While most autoantibody levels did not differ substantially between groups, moderate differences were observed for Chi3L1 and α-syn autoantibodies, particularly in individuals with mild cognitive impairment, suggesting a distinct antibody reactivity profile at early disease stages. Data were analyzed semi-quantitatively by categorizing samples according to autoantibody titers (“0” negative, “0+” very low, “+” low “++” moderate, “+++” high), see Methods for cut-offs. Stacked bar plots show the distribution of semi-quantitative autoantibody reactivity categories (0–+++) across diagnostic groups. Group differences were assessed using the Kruskal–Wallis test followed by pairwise comparisons. *p*-values were adjusted for multiple testing using the Benjamini–Hochberg false discovery rate procedure. Symbols indicating statistical significance (*p* values FDR-adjusted): * *p* < 0.05; ** *p* < 0.01 (see subfigure K). Color coding based on categorization of antigens outlined in Table 2. AD, Alzheimer’s disease; FTD, frontotemporal lobar neurodegeneration; PD, Parkinson’s disease; VD, vascular dementia; MCI, mild cognitive impairment; CTRL, control group. Aβ42, amyloid-beta 1–42 peptide; Tau441, full-length tau protein; α-syn, alpha-synuclein; FUS, fused in sarcoma; TDP-43, TAR DNA-binding protein 43; VSNL1, visinin-like protein 1; NFL, neurofilament light chain; MBP, myelin basic protein; TREM2, triggering receptor expressed on myeloid cells 2; NSE, neuron-specific enolase; Chi3L1, chitinase-3-like protein 1; GFAP, glial fibrillary acidic protein; MCP-1, monocyte chemoattractant protein-1; NRGN, neurogranin.

Relative serum levels of multiple Aabs in each group are presented in Figure 2. All statistical test *p*-values can be found in Table 4. Antibody levels against 12 of the 14 antigens did not differ between patient and control groups. Although anti-Chi3L1 and α-syn levels showed differences across groups, these findings were not corrected for multiple testing and should be interpreted cautiously. Future studies with larger cohorts and correction for multiple comparisons are needed to confirm these exploratory results. Importantly, significant difference was observed at the Chi3Li Aabs levels using a global Kruskal–Wallis test to assess differences between groups (*p* = 0.0192 after FDF correction for 14 tests). Post hoc analysis with the Mann–Whitney group comparison test followed by Benjamini–Hochberg false discovery rate correction revealed significantly elevated levels of anti-Chi3li in patients with MCI compared to AD (*p* = 0.001), PD (*p* = 0.0210), or the control group (*p* = 0.0210) (Table 5). Across several biomarkers, the largest effect sizes were consistently observed in comparisons involving the MCI group. In particular, Chi3L1 showed moderate differences between MCI and AD, controls, PD, and VD (effect size 0.33–0.47); α-syn also differed between MCI and both AD and control groups (effect size 0.29–0.42), and TREM2 differed between MCI and PD (effect size 0.36); PD and VD, AD and controls (effect size 0.32–0.55). This pattern suggests that the MCI cohort exhibits a distinct antibody reactivity profile relative to other diagnostic groups. Several comparisons involving the PD group showed moderate-to-large effect sizes, particularly for TREM2 and Chi3Li (effect size 0.36–0.55), despite the relatively small PD sample size. In contrast, other biomarkers showed minimal differences involving PD (effect size < 0.1), suggesting biomarker-specific patterns rather than random variability.

## 4. Discussion

In the present study, a panel of protein biomarkers of NDDs which are broadly involved in the specific or general pathologies present was extensively explored. Aab levels were measured across patients with dementia with various underlying pathologies, and healthy controls. We analyzed Aabs against the following sets of proteins: proteins reflecting an underlying pathology (tau, Aβ42, TDP43, α-syn, FUS); those related to myelin damage and axonal degeneration (NFL, MBP); those related to microglial and astrocyte activation (TREM2, Chi3Li); those related to synaptic loss (NRGN); and those associated with myelin damage (MBP), neuronal injury (NSE), disturbed calcium homeostasis (VSNL1), and neuroinflammation (MCP-1). Aabs were detected against all 14 antigens across all dementia groups and healthy controls. Interestingly, significantly higher levels of anti-Chi3Li Aabs were found in MCI patient group compared to patients with AD, PD, VD, and healthy controls. Chi3Li is a secreted glycoprotein involved in multiple pathophysiological processes, including inflammation, macrophage polarization, apoptosis, and carcinogenesis [38]. Chi3Li is expressed across various cell types such as macrophages, neutrophils, tumor cells, inflammatory cells, vascular smooth muscle cells, and central nervous system cells such as microglia, astrocytes, and neurons. This broad cellular distribution highlights the fundamental role of Chi3Li in regulating brain homeostasis and pathological processes [39]. Its expression is significantly upregulated in a variety of inflammatory and immunological disorders including cancer, AD, and atherosclerosis. During the progression of NDDs, Chi3Li contributes to pathological processes by activating the MAPK and NF-κB signaling pathways. This activation promotes Aβ accumulation and neuronal inflammation through the receptor for advanced glycation end products (RAGE) in both astrocytes and neurons. In neuronal cells specifically, Chi3Li further exacerbates neurotoxicity by upregulating the expression of CD14 and Toll-like receptor 4 (TLR4) via the same signaling cascades, ultimately leading to neuronal damage and cognitive decline. Recent studies have identified Chi3Li as a promising biomarker for clinical applications, with potential utility in disease diagnosis, prognosis, and in monitoring disease activity and severity [38,40]. According to Choi et al., a significant increase in mean plasma Chi3Li levels was observed in early AD patients compared with both control subjects and MCI patients. No significant difference was found between MCI patients and controls [41]. We could hypothesize that Aabs directed to the Chi3Li may be a first-line immune response. This possibility suggests that the immune system may initially recognize rising Chi3Li as a marker of emerging neuropathological changes and attempt to counteract its increase through humoral mechanisms. Chi3Li Aabs could potentially reflect the transition from preclinical stage to early symptomatic AD. Furthermore, investigating these Aabs may provide insight into the dynamics of immune activation in AD, reveal novel biomarkers for disease onset, and shed light on whether Chi3Li itself contributes to early pathology or whether it is a downstream consequence of an ongoing pathological process. Our findings present the first evidence of anti- Chi3Li Aabs in the plasma of patients with dementia and indicate altered levels in MCI relative to advanced dementia. Nevertheless, the biological role of the detected Aabs remains uncertain. Although natural Aabs have been proposed to contribute to immune homeostasis and receptor modulation, the present study did not assess the functional properties of the detected antibodies. Hence, any interpretation regarding potential immunomodulatory or protective effects should be properly addressed in future mechanistic studies. Diagnostic and prognostic utility of Chi3Li Aabs should require independent replication cohorts and longitudinal data to draw any conclusions.

The detection of Aabs in healthy controls should be interpreted within the framework of physiological Aabs networks. Naturally occurring Aabs are commonly present in healthy individuals and are considered an integral component of immune homeostasis [42]. These antibodies participate in immune surveillance processes, including the clearance of apoptotic cells, cellular debris, and misfolded or aggregated proteins, and may reflect normal regulatory immune mechanisms [43]. The role of immunity involvement in NDDs seems to have gained larger importance over the past years. Until recently, most of the research on Aabs related to the NDDs was focused on natural Aβ peptide-reactive antibodies with first Aabs against Aβ protein discovered more than 30 years ago [44]. Since then, numerous studies have been exploring different aspects of the Aabs’ characterization, isotype, antigenic specificities and affinities, and their role in human body. The anti-seeding and potential to slow aggregation and cytotoxicity of Aabs against α-syn was proposed [45,46,47]. Intravenous immunoglobulin products were tested as a potential treatment strategy in combatting the progression of NDDs due to the critical involvement of the immune system in the pathobiology of NDDs, and antibody-based therapies are under close investigation as a promising disease-modifying strategy. Natural polyclonal immunoglobulins directed against Aβ peptides and tau proteins have been debated as potential passive immune therapy amongst other candidates, and active immunization has been tested confirming clinical benefits in human studies [48,49,50]. The presence of anti-Aβ Aabs and anti-αSyn Aabs is ubiquitous in the blood and CSF of both healthy individuals and patients with AD or PD. This finding underscores the importance of their epitopes, highlights epitope relevance, and describes dynamic changes in anti-Aβ antibodies in AD patients. Levels of Aabs targeting the amino terminus of Aβ were shown increased, and those targeting the mid-domain of Aβ decreased in both CSF and plasma in AD patients [19,51]. The identification of anti-tau Aabs in peripheral circulation represents a significant advancement in the field. Their presence from early childhood implies that their generation is independent of exogenous antigen exposure, suggesting a role as components of the innate immune repertoire [52]. This further supports their potential involvement in the regulation of tau homeostasis under both physiological and pathological conditions [25]. Previously, we showed lower levels of anti-tau Aabs in AD patients in comparison to healthy controls in our laboratory [53]. This finding was not confirmed in the present study. A possible explanation could be the relatively small number of healthy participants in the current study, limiting statistical power. In addition, despite extensive investigation, it remains unclear whether peripheral tau Aab levels are altered in AD and MCI patients [15].

The humoral immune response targeting myelin sheath proteins was investigated in AD previously with the aim of identifying potential early biomarkers associated with memory impairment and the characteristic neuropathological changes of the disorder [54]. Significantly elevated serum titers of IgG Aabs were detected in AD patients against all assessed myelin-related antigens, such as myelin oligodendrocyte glycoprotein (MOG), myelin-associated glycoprotein (MAG), and MBP and proteolipid protein (PLP), when compared to healthy controls [55]. In our laboratory, a decreased level of heavy neurofilament in the serum of patients with AD and elevated intrathecal synthesis in patients with AD and multiple sclerosis was detected; levels of NFL were found unchanged which is consistent with our present findings [53,56,57].

TREM2 is a lipid-binding receptor which is expressed in macrophages. TREM2 macrophages include microglia in the central nervous system; macrophages in the liver, skin, gut, adipose tissue, and tumors; and osteoclasts in the bone [58]. TREM2 has been implicated in neuroprotection across various NDDs [59,60,61,62]. As AD progresses, homeostatic microglia gradually transform into disease-associated microglia (DAM) with distinct functional and transcriptional features. DAM help by clearing Aβ plaques and protecting nearby neuronal tissue and is dependent on TREM2. Rare loss-of-function mutations in gene for TREM2 raise the risk of AD. TREM2 also enhances mTOR signaling and improves microglia’s metabolic capacity [63]. Preclinical studies strongly support a protective role for microglial TREM2 in relation to Aβ, as well as Aβ-induced tau pathology. Following the identification of the TREM2 Arg47His variant as a major genetic risk factor for late-onset AD [64,65], extensive work in Aβ mouse models has further been done. Loss of TREM2 function and/or expression of the human Arg47His variant enhanced the seeding and spreading of tau pathology in regions close to Aβ plaques, indicating that TREM2 normally constrains Aβ-driven propagation of tau. Moreover, mice lacking the TREM2 gene exhibited reduced neuroinflammation and were protected from tau-mediated cell loss [66]. Recent studies further highlight TREM2’s protective role in mitigating neurodegeneration and neuroinflammation in PD [67,68]. Monoclonal agonistic anti-TREM2 antibodies have been developed to enhance protective TREM2 signaling in mouse microglia [69].

In a study by Okuzono, et al. [70], activating TREM2 using an agonistic antibody (Hyb87) led to a specific gene expression in microglia. TREM2 activation decreased with the progression of AD and, conversely, higher TREM2 activation was associated with protective pathways such as anti-apoptotic signaling, immune regulation, and cytoskeletal remodeling. In the present study, high seroprevalence of TREM2 Aabs in healthy controls compared with the PD and FTD groups was shown. It the light of these findings, naturally occurring anti-TREM2 Aabs in healthy individuals could potentially contribute to maintaining microglial homeostasis or neuroprotective signaling. Future studies assessing the functional properties of circulating anti-TREM2 Aabs, such as their ability to induce downstream TREM2 signaling or transcriptional responses in microglial models, would help to clarify whether these antibodies exhibit agonistic activity.

Our result suggests another important fact: concomitance of pathologies within clinical syndrome/diagnosis. Anti-α-syn Aabs were higher in MCI patients compared to AD and healthy controls. Even though this result does not reach statistical significance, it is strongly suggesting clinical relevance. α-syn pathology is present in 30–50% of AD patients, and its interactions with tau proteins may further exacerbate pathological changes in AD as has been showed previously [71]. Furthermore, patients with autopsy-confirmed Lewy body variants of AD experienced more rapid cognitive decline and higher mortality rates [72] and recent research has reported faster and more pronounced cognitive dysfunction with co-expression of tau with α-syn in the gut, suggesting that the interaction between α-syn and tau may further influence pathological changes in AD [73]. A recent study investigated both the affinities of IgGs and levels of IgGs, IgMs and IgAs towards α-syn, Aβ, and tau across AD, PD, and DLB patients. They found lower levels of high-affinity anti-Aβ and anti-α-syn IgGs in AD and PD patients compared to healthy controls [74]. There is not much data in the literature regarding α-syn levels in MCI patients; one longitudinal study evaluated levels of α-syn in CSF of AD patients. Interestingly, MCI-AD (MCI patients who developed AD in a 2 y follow-up, and with a short duration of symptoms prior to inclusion in the study </= 2 y), had considerably higher mean CSF α-syn levels compared to patients with a longer symptom duration. Variations in Aab levels suggest a complex interplay amongst immune responses in healthy and pathological states, potentially uncovering underlying pathophysiological changes which occur years before clinical diagnosis is developed. Limitations of this study remain present. Aab reactivity was analyzed using semi-quantitative OD categories derived from assay sensitivity thresholds (LOD and LOQ). While this approach facilitates standardized comparison across antigens with differing signal ranges, it may reduce the sensitivity necessary to detect subtle quantitative differences in Aab levels. Despite a wide selection of antigens and a cohort comprising the total of 159 study participants, some of the disease groups and a control group were relatively small and could affect the robustness of statistical analysis. To address this issue, effect sizes for Chi3Li, TREM2, and α -syn were calculated. Interestingly, the most pronounced differences in Aab reactivity were consistently observed in comparisons involving the MCI group. This finding may reflect immunological changes occurring at early stages of neurodegeneration, preceding the development of clinically established dementia syndromes. Although the PD cohort was relatively small, comparisons involving PD showed consistent effect-size patterns. Biomarkers such as TREM2 and Chi3Li demonstrated moderate differences relative to other diagnostic groups, whereas other markers showed minimal effects. This pattern suggests biomarker-specific differences rather than random variation related to sample size. Differences in age and sex distribution were observed between diagnostic groups. As both factors may influence biomarker levels, these demographic differences could partially contribute to the observed variability in Aab reactivity across groups. The observed difference in sex distribution between diagnostic groups was primarily driven by the FTD cohort, which contained a higher proportion of male participants. Importantly, the FTD group did not contribute to the biomarker comparisons with statistically significant differences (Table 4). Therefore, it is unlikely that the observed sex imbalance substantially influenced the key findings of the study. The AD group was on average older than several other diagnostic groups, particularly MCI and FTD. Since age may influence biomarker levels, this difference represents a potential confounding factor. However, the observed differences in Chi3L1 were not restricted to comparisons involving the AD group. Additionally, the MCI group differed significantly from other diagnostic groups that did not differ in age, suggesting that age alone is unlikely to account for the observed biomarker differences.

## 5. Conclusions

Our findings provide the first evidence for the presence of anti-Chi3Li Aabs in the plasma of patients with dementia and suggest altered levels in patients with MCI compared to the ones with advanced dementia stages. Emerging evidence indicates that inflammation, immune mechanisms, and vascular risk factors all play key roles in NDDs, and our results support the widely discussed involvement of autoimmunity in the pathogenesis of NDDs. A carefully designed collection of biomarkers such as Aabs may improve predictive and diagnostic accuracy in the early stages of disease, which is particularly relevant in the current era of emerging disease-modifying therapies. The current study was not designed to evaluate diagnostic or prognostic utility, and longitudinal studies with independent replication cohorts are needed to replicate these results in larger cohorts to explore biomarker utility and exact modulatory functions of the anti-Chi3Li Aabs in prodromal phases of the disease.

## Figures and Tables

**Table 1:** Characteristics of 159 participants enrolled in this study.

	AD	MCI	PD	FTD	VD	Control
	(*n* = 58)	(*n* = 28)	(*n* = 10)	(*n* = 36)	(*n* = 15)	(*n* = 12)
Age, years	74.3 (7.9) *	69.2 (10.3)	72.3 (7.6)	67.8 (9.5)	66.8 (12.0)	67.6 (18.3)
Sex, Female, %	53.8	57.1	50.0	20.0 †	33.3	44.4
MMSE	20.4 (5.05)	22.9 (7.95)	24.0 (2.39)	21.0 (7.32)	25.0 (4.0)	NA

MMSE—the Mini-Mental State Examination, NA—not available. Values are mean (SD) or %. AD, Alzheimer’s disease; MCI, mild cognitive impairment; PD, Parkinson’s disease; FTD, frontotemporal lobar neurodegeneration; VD, vascular dementia; Control, cognitively healthy control group. Age missing for *n* = 17; sex missing for *n* = 15 out of the total number of participants; values reported based on available data. * statistically significantly different from FTD, VD, control group. † statistically significantly different from AD and MCI.

**Table 2:** Recombinant human antigens used for the assays, their functional relevance and source information.

Antigen	Full Name/Description	Pathological or Functional Relevance	Producer	Catalog Number
AD pathology				
Tau441	Full-length tau protein (441aa isoform)	Microtubule-associated protein; key pathological feature of tauopathies and AD	In house production *	N/A
Aβ42	Amyloid-beta 1–42 peptide	Core component of amyloid plaques in AD	Abcam, Cambridge, UK	ab120301
Differential diagnosis/mixed dementia detection				
α-syn	Alpha-synuclein	Major component of Lewy bodies; hallmark of PD and related synucleinopathies	Sigma Aldrich, St. Louis, MO, USA	S7820
FUS	Fused in sarcoma	RNA-binding protein implicated in ALS and FTD	Sigma-Aldrich, St. Louis, MO, USA	APREST86697
TDP43	TAR DNA-binding protein 43	Pathological inclusion protein in FTD and ALS	Abcam, Cambridge, UK	ab41970
Dendrite and axon degeneration				
VSNL1	Visinin-like protein 1	Neuronal calcium sensor; associated with synaptic loss and cognitive impairment	MyBioSource, San Diego, CA, USA	MBS286167
NFL	Neurofilament light chain	Structural axonal protein; biomarker of axonal degeneration across NDDs	MyBioSource, San Diego, CA, USA	MBS2010106
MBP	Myelin basic protein	Major structural component of the myelin sheath; biomarker of myelin damage and demyelination, particularly in inflammatory diseases and NDDs	Abcam, Cambridge, UK	ab4361
Microglial and astrocytic activation				
TREM2	Triggering receptor expressed on myeloid cells 2	Marker of microglial activation; linked to AD and neuroinflammation	Abbexa, Cambridge, UK	abx655342
GFAP	Glial fibrillary acidic protein	Intermediate filament protein; indicator of astrocyte activation and gliosis	Antibodies online, Aachen, Germany	ABIN368852
Chi3Li (YKL-40)	Chitinase-3-like protein 1	Secreted glycoprotein; astrocytic and microglial activation marker	Acro Biosystems, Newark, DE, USA	CH1-H5228
NSE	Neuron-specific enolase	Glycolytic enzyme, marker of neuronal injury and neurodegeneration	Thermofisher, Erlangen, Germany	RP-75668
MCP-1 (CCL2)	Monocyte chemoattractant protein-1	Chemokine mediating leukocyte recruitment and blood–brain barrier disruption	Abbexa, Cambridge, UK	abx068048
Synaptic loss				
NGRN	Neurogranin	Marker of synaptic dysfunction and cognitive decline	MyBioSource, San Diego, CA, USA	MBS1340607

* Tau441 was produced in-house using recombinant expression and purification methods established in our laboratory [25]. All other proteins were recombinant human antigens (≥95% purity).

**Table 3:** Titer scoring.

Score	Definition	Interpretation
0	ODc + increase of ≤0.05 OD	No detectable reactivity
0+	ODc + increase of >0.05 ≤ 0.1 OD	Reactivity above LOD
+	ODc + increase of >0.10 ≤ 0.2 OD	Reactivity above LOQ
++	ODc + increase > 0.20 ≤ 1.00 OD	Moderate reactivity
+++	ODc + increase of >1.00 OD	High reactivity

Optical density (OD) of samples was categorized to distinct classes in relation to OD of uncoated control well (ODc).

**Table 4:** Differences between autoantibody levels among groups of patients and healthy controls—uncorrected *p* values.

	TREM2	MCP-1	α-syn	NFL	MBP	NSE	TDP-43	FUS	Aβ	NGRN	Chi3Li	GFAP	Tau441	VSNL1
global KW-test	0.0576	0.4772	0.0500	0.3564	0.4428	0.7514	0.2185	0.3076	0.9603	0.8951	0.0014	0.2651	0.6510	0.6139
AD vs. FTD	0.1098	0.6024	0.2938	0.9773	0.2741	0.5744	0.4813	0.9231	0.6349	0.7354	0.0618	0.4870	0.7385	0.9314
AD vs. PD	0.0078	0.4611	0.8898	0.5756	0.1542	0.6130	0.6521	0.6050	0.7638	0.7323	0.7634	0.8213	0.2887	0.5440
AD vs. VD	0.7070	0.5830	0.1493	0.0845	0.1343	0.2302	0.5205	0.5679	0.4552	0.2804	0.2295	0.7384	0.8833	0.2843
AD vs. MCI	0.7845	0.2119	0.0077	0.3096	0.8209	0.6412	0.0147	0.0624	0.9374	0.9223	0.0001	0.0191	0.2547	0.3814
AD vs. contr	0.3905	0.1126	0.2437	0.5120	0.6550	0.6655	0.1210	0.5089	0.7693	0.9083	0.6661	0.8392	0.3351	0.3469
FTD vs. PD	0.1382	0.6640	0.6331	0.6608	0.5530	0.4091	0.9528	0.5875	0.5728	0.9224	0.1400	0.8687	0.2556	0.6686
FTD vs. VD	0.4216	0.4242	0.6216	0.1255	0.5711	0.4613	0.8628	0.6554	0.7236	0.4112	0.6036	0.8223	0.6523	0.3162
FTD vs. MCI	0.1605	0.4655	0.1757	0.4140	0.4101	0.9553	0.0948	0.0878	0.6569	0.6783	0.0891	0.1207	0.2623	0.4881
FTD vs. contr	0.0873	0.2393	0.1108	0.5555	0.6538	0.4440	0.3349	0.5967	0.9390	0.7202	0.1650	0.5127	0.2726	0.4338
PD vs. VD	0.0290	0.3631	0.4094	0.3162	1.0000	0.1879	1.0000	0.5063	0.4421	0.7323	0.2053	0.9538	0.4161	0.2814
PD vs. MCI	0.0252	0.9397	0.1513	0.2678	0.1309	0.4684	0.2602	0.7831	0.8331	0.8307	0.0036	0.2187	0.7939	0.9160
PD vs. contr	0.0097	0.5904	0.3941	0.7947	0.1736	0.9447	0.4401	0.2785	0.6437	0.8873	0.8858	0.7327	0.8512	0.8584
VD vs. MCI	0.6233	0.2024	0.5245	0.0338	0.1448	0.4837	0.2577	0.0465	0.4789	0.2082	0.0306	0.1009	0.5350	0.1102
VD vs. contr	0.3031	0.1162	0.0602	0.5359	0.2522	0.2135	0.5512	0.9798	0.7197	0.2724	0.2095	0.6495	0.4316	0.1411
MCI vs. contr	0.6175	0.5614	0.0081	0.2685	0.8880	0.4777	0.7205	0.0359	0.6513	0.9599	0.0042	0.0667	1.0000	0.7407

Abbreviations: AD, Alzheimer’s disease; FTD, frontotemporal dementia; PD, Parkinson’s disease; VD, vascular dementia; MCI, mild cognitive impairment; contr, controls. Colors: 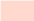 0.05 ≤ *p* < 0.1, 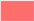 0.01 ≤ *p* < 0.05, 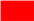 0.001 ≤ *p* < 0.01, 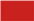 *p* < 0.001.

**Table 5:** Differences between Chi3Li autoantibody levels among group of patients and healthy controls—*p*-values after correction for multiple testing.

	AD	FTD	PD	VD	MCI
FTD	0.1855				
PD	0.8179	0.3001			
VD	0.3130	0.7545	0.3130		
MCI	**0.0010**	0.2227	**0.0210**	0.1149	
contr	0.7685	0.3094	0.8858	0.3130	**0.0210**

Abbreviations: AD, Alzheimer’s disease; FTD, frontotemporal dementia; PD, Parkinson’s disease; VD, Vascular dementia; MCI, mild cognitive impairment; contr, controls. *p*-values less than 0.05 were considered statistically significant and are presented in boldface. Colors: 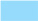 0.01 ≤ *p* < 0.05, 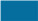 *p* < 0.001.

## Data Availability

The data presented in this study are available on request from the corresponding author due to privacy and confidentiality.
